# Innovative Peptide Therapeutics for SARS-CoV-2: Design, Docking, and Functional Analysis

**DOI:** 10.5812/ijpr-160762

**Published:** 2026-02-15

**Authors:** Samaneh Karimkhanilouei, Saeid Ghorbian, Sanaz Mahmazi, Changiz Ahmadizadeh, Keivan Nedaei

**Affiliations:** 1 Department of Biology, Ah.C., Islamic Azad University, Ahar, Iran; 2 Department of Biology, Ta.C., Islamic Azad University, Tabriz, Iran; 3 Department of Biology, Za.C., Islamic Azad University, Zanjan, Iran; 4 Department of Medical Biotechnology, Faculty of Medicine, Zanjan University of Medical Sciences, Zanjan, Iran

**Keywords:** SARS-CoV-2, Inhibitory Peptide, Peptide Design, Molecular Docking, Molecular Dynamics Simulation

## Abstract

**Background:**

The continuous emergence of severe acute respiratory syndrome coronavirus 2 (SARS-CoV-2) variants necessitates the rapid development of novel therapeutics, particularly those targeting conserved viral proteins. Peptide-based drugs offer high specificity and low toxicity, making them ideal candidates.

**Objectives:**

This study employed an integrated computational approach, combining structural biology, molecular docking, and molecular dynamics (MD) simulations, to design and evaluate novel peptide analogs targeting three key proteins of SARS-CoV-2: the Spike (S) protein, RNA-dependent RNA polymerase (RdRp), and nucleocapsid (N) protein.

**Methods:**

The first step involved preparing a dataset containing anti-SARS-CoV-2 peptides using the DRAVP database and a literature survey. Then, the best inhibitory peptides were screened using the AVPPred tool, and analogous peptides were designed based on the selected lead peptide. The designed peptides were then investigated in terms of their structure, physicochemical properties, and antiviral potency. Additionally, molecular docking, performed using the specialized nCoVDock2 server, showed that all designed analogs exhibited highly favorable binding. Specifically, the best-performing analogs achieved remarkable docking scores in the range of -200 to -300 a.u. (arbitrary units), indicating a strong predicted relative binding affinity for their respective targets. The top-ranked complexes were then subjected to 100 ns explicit solvent MD simulations.

**Results:**

Our findings suggest that peptide W is the most effective analogue for inhibiting S protein, achieving a relative docking score of -303.41 a.u., in contrast to the -284.12 a.u. relative docking score of the EK1 lead peptide. Regarding the inhibition of RdRp protein, the top newly designed analogue is peptide A5, which has a relative docking score of -187.36 a.u., compared to the score of -121.3 a.u. for lead peptide 5, respectively. The leading novel analogue for inhibiting the N protein is A7, which has a relative docking score of -317.69 a.u., surpassing the relative docking score of -255.48 a.u. for Plectasin. The MD results confirmed the high dynamic stability of the W (targeting S protein) and A5 (targeting RdRp) complexes, demonstrating low Root Mean Square Deviation (RMSD) and maintaining critical hydrogen bonds and hydrophobic interactions throughout the trajectory.

**Conclusions:**

The use of bioinformatics algorithms to develop engineered peptides with high affinity for SARS-CoV-2 virulence proteins offers a promising outlook for peptide-based therapies against SARS-CoV-2. It also presents a promising approach for developing therapeutic methods against other viral diseases. Furthermore, these computational insights lay the groundwork for subsequent in vitro and in vivo validation studies to ascertain the therapeutic efficacy and safety profiles of the identified peptide candidates.

## 1. Background

The COVID-19 pandemic, which began in late 2019, has placed a significant strain on global healthcare systems and economies. The etiological agent of this disease, severe acute respiratory syndrome coronavirus 2 (SARS-CoV-2), is a member of a large family of single-stranded RNA viruses that can infect a broad range of species, including birds, mammals, and especially humans. This spherical, enveloped virus contains a positive-sense RNA genome (5' to 3' direction), allowing it to function directly as messenger RNA (1-3). COVID-19 can affect the respiratory and digestive systems, leading to a cytokine storm that causes tissue damage, systemic failure, and, in severe cases, death (4).

Although vaccines have been developed to prevent infection, the high mutation rate of SARS-CoV-2 and the potential adverse effects underscore the critical need for effective antiviral drugs (5, 6). In this regard, data obtained from genomic and structural analysis of SARS-CoV-2 have opened new avenues for rational drug design (7). The SARS-CoV-2 genome encodes various non-structural proteins (NSPs) and several structural proteins. The structural proteins, including the nucleocapsid protein (N), membrane protein (M), spike protein (S), and envelope protein (E), are crucial for viral function (2, 3). Among these, the S and N proteins are considered highly suitable targets for drug discovery due to their vital roles in the viral life cycle (3, 8).

The N protein is involved in nucleocapsid formation and assembly. It acts as a virulence factor by inducing lung inflammation and suppressing the immune response through the activation of cyclooxygenase-2 (COX-2) and inhibition of type I interferon, respectively (9, 10). Furthermore, this protein can interfere with the cell cycle and proteasome-dependent degradation of viral proteins. Conversely, its interaction with heterogeneous nuclear ribonucleoprotein (hnRNPA1) promotes viral RNA synthesis. The N protein also possesses significant immunogenicity (11).

The spike (S) glycoprotein facilitates the entry of SARS-CoV-2 into host cells by binding to the angiotensin-converting enzyme 2 (ACE2) receptor. This homotrimeric protein consists of two subunits, S1 and S2. The receptor-binding domain (RBD) within the S1 subunit mediates the interaction with host cells. As the S-glycoprotein is unique to the virus, inhibiting it can effectively block viral entry, making it a promising therapeutic target (7, 12).

In addition to the structural proteins, SARS-CoV-2 also encodes several non-structural proteins that facilitate virus replication and regulate the host immune system. These include papain-like protease (nsp3), main chymotrypsin-like protease (3CL protease, nsp5), RNA-dependent RNA polymerase (RdRp, nsp12), helicase (nsp13), and exoribonuclease (nsp14). RdRp (nsp12) is a particularly crucial enzyme for viral RNA replication. The development of drugs that inhibit key viral functions, such as those of proteases and polymerases, has represented a significant advancement in antiviral research. This breakthrough has led to the creation of many successful antiviral medications for combating HIV and hepatitis C virus (HCV) and has paved the way for targeting various other virus families. Consequently, the RdRp has been extensively studied as a potential target for COVID-19 treatment (13).

Peptides have emerged as a powerful, specific, and safe class of therapeutic agents in modern research. They possess highly desirable characteristics, including low toxicity, high specificity, and minimal side effects. Furthermore, peptides have demonstrated effectiveness as viral inhibitors, with notable examples against viruses such as Zika, West Nile, and HIV (14, 15).

Computer-aided drug design strategies are indispensable tools for the efficient development of peptide-based therapeutics. These methods, which include molecular docking and structural modeling, enable the rational design of peptides with strong affinities for their targets. Access to databases containing sequences, physicochemical properties, and known effects of existing antiviral peptides can significantly accelerate the design process and enhance its precision (16).

## 2. Objective

This study presents a computational framework for the rational design of novel peptide inhibitors targeting key SARS-CoV-2 proteins. We address the critical need for effective COVID-19 therapeutics by utilizing a data-driven approach. Specifically, a comprehensive collection of anti-SARS-CoV peptides was initially sourced from DRAVP, a dedicated database of antiviral peptides and proteins. From this collection, peptides associated with the S, N, and RdRp receptors were selected and used as a basis to design novel sequences with potentially improved inhibitory efficiency. The findings from this work provide a set of promising lead candidates and offer valuable insights into the development of effective, peptide-based medications against COVID-19.

## 3. Methods

### 3.1. Preparation of Anti-SARS-CoV-2 Peptide Dataset and Selection of Lead Peptides

A comprehensive dataset of anti-SARS-CoV-2 peptides was compiled through extraction from the DRAVP database (dravp.cpu-) (17) and a systematic literature review. Subsequently, the AVPPred tool (crdd.osdd.net) was employed to identify suitable lead peptides based on antiviral motifs, amino acid composition, and physicochemical property models.

### 3.2. Determination of Binding Hotspots

The crystal structures of SARS-CoV-2 S (PDB ID: 7C53), N (PDB ID: 6M3M), and RdRp (PDB ID: 7BV2) proteins were used as target receptors, along with the crystallographic forms of lead peptide complexes and their corresponding receptor interactions, which were retrieved from the RCSB Protein Data Bank (rcsb.org). The Contact Finder web server (bioinf.modares) was subsequently utilized to identify key interactions between target receptors and lead peptides. PDBsum software (ebi.ac.uk) was employed to provide comprehensive overviews of each 3D structure registered in the protein database, and schematic diagrams of amino acid interactions were generated to determine critical residues involved in peptide-receptor binding. Due to the absence of crystallographic structures for N and RdRp protein complexes, relevant literature was referenced to identify amino acids participating in complex formation (15, 18).

### 3.3. Analogue Design and Structural Modeling

Following the identification of key amino acids and interactions between lead peptides and their target receptors, strategic mutations were introduced to enhance secondary structure stability and strengthen receptor interactions. The three-dimensional and secondary structures of the designed analogues for each lead peptide were subsequently determined. To model the three-dimensional structures and generate PDB files, we utilized ColabFold v1.5.5 (colab.research.google), a user-friendly platform that leverages the powerful AlphaFold2 and AlphaFold-Multimer models. For each peptide, five models were computed, and the most stable model (model 1) was selected for further analysis. The accuracy of the modeled structures was validated using metrics such as predicted local distance difference test (pLDDT) and sequence coverage.

Secondary structure analysis of the designed analogues was performed using the online Secondary Structure Prediction tool (cib.cf.ocha.ac). This comprehensive tool predicts alpha-helices and beta-strands from amino acid sequences by integrating three well-established and complementary methods: Chou-Fasman, Garnier-Osguthorpe-Robson (GOR), and Neural Networks. The consensus predictions from these methods provided a robust basis for evaluating the impact of the introduced mutations on the peptide's secondary structure.

### 3.4. Assessment of Physicochemical Properties and Toxicity of Designed Analogues

The physicochemical properties of designed analogues, including molecular weight, net charge at neutral pH, isoelectric point, and grand average of hydropathicity (GRAVY) (19), were analyzed using the Novoprolabs web server tool (novoprolabs.com). In addition, the parameters of acidity, basicity or neutrality, and hydrophobicity were assessed using the Biosyn online tool (biosyn.com).

Toxicity assessment of designed peptides was conducted using the ToxinPred web-based tool (iiitd.edu). This platform enables toxicity prediction and provides options for identifying mutations that can modulate peptide toxicity levels. The tool generates comprehensive lists of all potential mutants for specified peptides (including all possible single mutations) and estimates the toxicity of each variant alongside essential physicochemical properties such as hydrophobicity, charge, and isoelectric point (pI). This functionality allows users to identify and modify specific residues that may significantly reduce peptide toxicity (20).

### 3.5. Energy Minimization and Model Validation

Energy minimization was performed using the Amber force field implemented in HyperChem software to obtain thermodynamically stable structures. The topology of the lowest energy conformation was saved in PDB format for subsequent analyses.

### 3.6. Molecular Docking

Molecular docking studies of lead peptides and designed analogues with S, N, and RdRp proteins were performed using the nCoVDock2 server (ncovdock2.schanglab) (21). This platform supports docking of small molecules, peptides, and antibodies. The results, reported as relative docking scores (in arbitrary units, a.u.), were used to measure binding affinity. Interaction profiling was subsequently analyzed using LigPlot+ (22).

### 3.7. Molecular Dynamics Simulations

Molecular dynamics (MD) simulations were conducted using GROMACS 2022.4 with the CHARMM36 force field to evaluate the stability and binding dynamics of peptide-receptor complexes. Initial docked structures were solvated in TIP3P water boxes, neutralized with counterions (Na⁺/Cl⁻), and adjusted to physiological ionic strength (0.15 M NaCl). All systems underwent energy minimization using the steepest descent algorithm for 50,000 steps, followed by equilibration in the NVT ensemble (100 ps at 310 K using V-rescale thermostat) and the NPT (1 ns at 1 bar using Berendsen barostat) with positional restraints applied to heavy atoms.

Production runs were performed for 100 ns in the NVT ensemble using the Nose-Hoover thermostat, with a 2-fs time step, LINCS constraints for bond lengths, 1.2 nm cutoff for non-bonded interactions, and particle mesh Ewald (PME) method for long-range electrostatic calculations. Trajectory analyses included root mean square deviation (RMSD), root mean square fluctuation (RMSF), hydrogen bond occupancy (distance cutoff: 3.5 Å, angle cutoff: 30°), and binding free energy calculations using the MM-PBSA method implemented via g_mmpbsa. All analyses were performed using GROMACS utilities and visual molecular dynamics (VMD) software. Simulations were conducted in triplicate to ensure statistical reliability and reproducibility, following established protocols for viral protein-ligand systems.

## 4. Results

### 4.1. Dataset Preparation and Peptide Selection

A comprehensive dataset comprising 148 antiviral peptides specific to SARS-CoV-2 was initially assembled from the DRAVP database and literature sources. Following the removal of peptides with unidentified target proteins, 81 peptides with well-characterized receptors were retained for subsequent analysis. Since the peptide design approach in this study relies on structure-based considerations, identification of specific target receptors was essential for rational design strategies.

**Figure 1.**
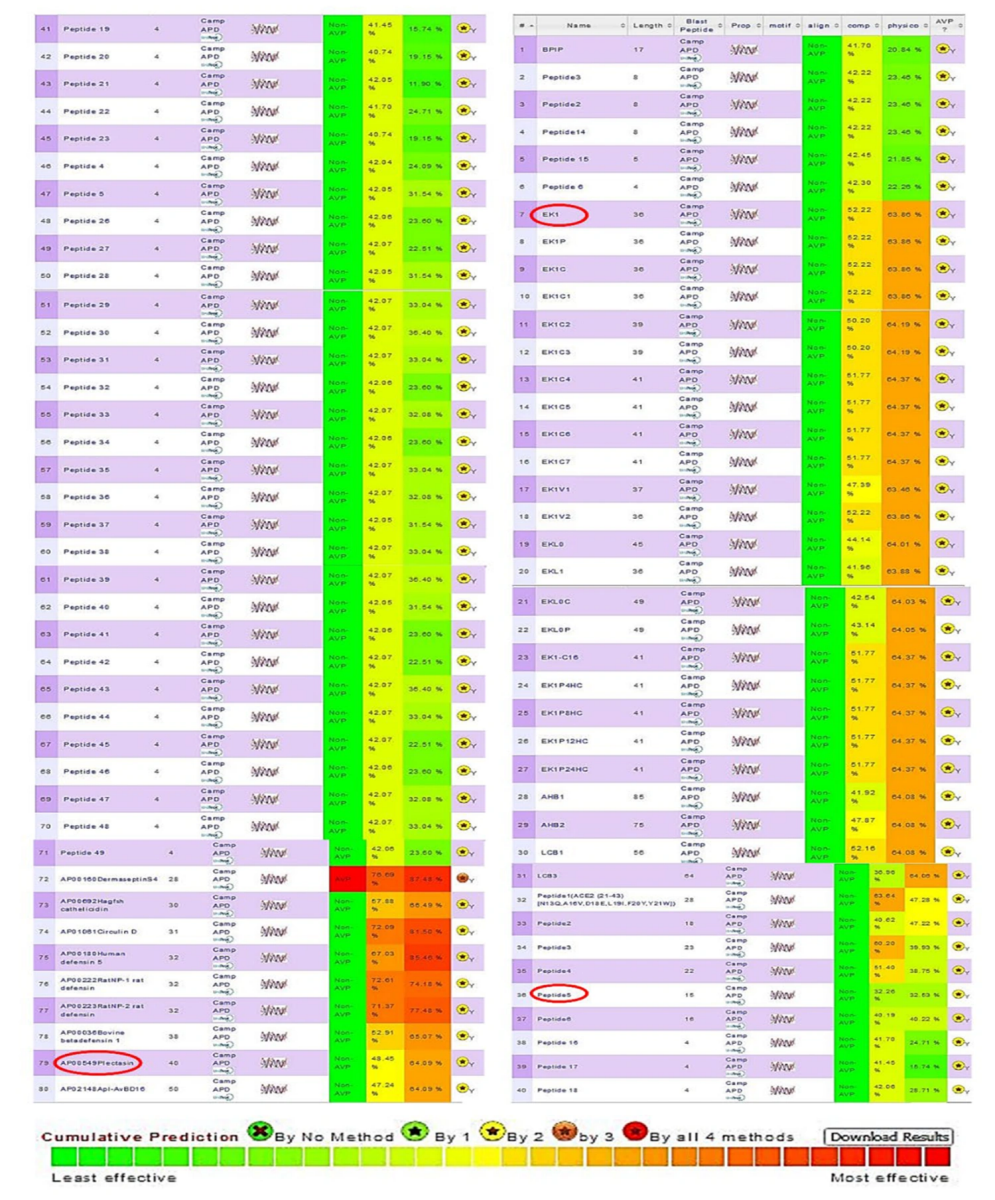
The output of the AVPPred tool for predicting the inhibitory effect of peptides related to the S, N, and RdRp receptors, where the selected peptides — EK1, Peptide 5, and Plectasin — are marked with a red box.

The 81 peptides underwent systematic screening using the AVPPred tool, which evaluates antiviral activity based on sequence motifs, amino acid composition, and physicochemical properties. This screening process focused on peptides targeting the three key SARS-CoV-2 proteins: Spike (S), RNA-dependent RNA polymerase (RdRp), and nucleocapsid (N) proteins. Based on the AVPPred predictions, three peptides demonstrated optimal inhibitory potential and were selected as lead compounds: EK1 for the S protein, peptide 5 for RdRp, and Plectasin for the N protein (Table 1 and Figure 1). 

**Table 1.** Information on the Selected Peptides as Lead Compounds for S, RdRp, and N Proteins

Peptide	Target Protein	Sequence	Number of Amino Acids	GRAVY	Theoretical pI (PH)	Charge at PH7	Hydrophobic (%)	Neutral (%)	Basic (%)	Acidic (%)
**EK1**	S	SLDQINVTFLDLEYEMKKLEEAIKKLEESYIDLKEL	36	-0.43	4.14	-5	44.44	13.89	13.89	27.78
**Peptide 5**	RdRp	ARKD	4	-2.53	10.09	1	25	50	0	25
**Plectasin**	N	GFGCNGPWDEDDMQCHNHCKSIKGYKGGYCAKGGFVCKCY	40	-0.7	7.64	0.9	10	17.5	32.5	40

Of the 81 peptides introduced, we selected the target peptides based on articles confirming their inhibitory activity through MD studies and emphasizing their significance. According to a literature survey, peptide 5, with ΔGbind of -17.41 kcal/mol, was identified as the best anti-SARS-CoV-2 peptide (15). Ultimately, we selected peptide 5 as the best lead peptide for designing RdRp analogues, as per the reviewed articles. Information about these lead peptides is also included in Table 1. The lead inhibitory peptide for the N protein was chosen based on a literature review. Plectasin was identified as the top peptide for targeting SARS-CoV-2 and as the lead peptide for analogue design, with a low docking score and a binding affinity of -3.4 kcal/mol (Table 1) (18). Similarly, the lead peptide for the S protein, EK1, was selected as a potent fusion inhibitor based on multiple studies that confirmed its broad and powerful inhibitory activity against SARS-CoV-2 and its variants by targeting the highly conserved HR1 region of the S2 subunit (23-25). This peptide was chosen as the lead for our analogue design due to its proven efficacy and structural stability (Table 1). 

### 4.2. Analysis of Key Amino Acid Interactions Between Lead Peptides and Target Proteins

**Figure 2.**
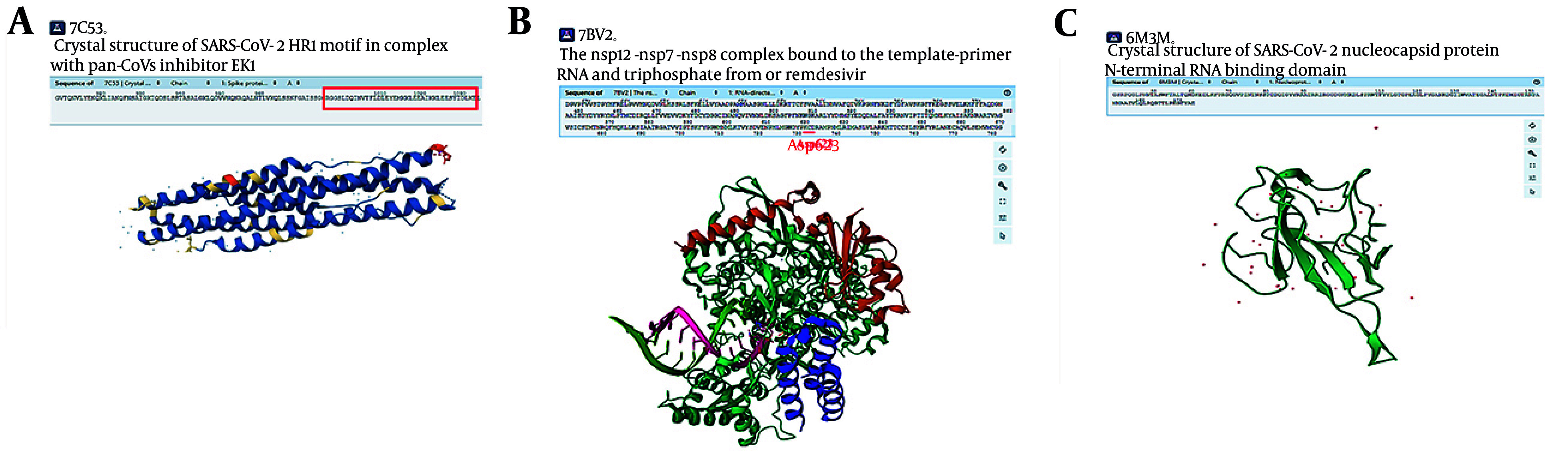
The crystallographic structure of receptors. A, the crystallographic structure of the EK1 peptide and the S protein receptor; the sequence of the EK1 peptide is marked with a red box; B, the RdRp receptor's crystallographic structure and the binding region of the lead peptide to the amino acid Asp623 of the receptor are marked with a red line; C, the crystallographic structure of the N protein.

The PDB structures of S (PDB ID: 7C53), RdRp (PDB ID: 7BV2), and N (PDB ID: 6M3M) receptors, along with the crystal structures of the Receptor Binding Domain in complex with lead peptides (Figure 2), were downloaded from the Protein Data Bank and RCSB database, respectively. The lead peptide 5-RdRp and Plectasin-N complex files were not accessible. Consequently, only the interactions reported in the literature review (15, 18) were analyzed (Figure 2B and C).

Interactions between the S protein receptor and EK1 peptide were investigated based on the literature review (Table 2) (25) and the PDBsum tool (Table 3), and the important amino acids involved in the interaction were identified. As shown in Table 3, 17 amino acids from the S receptor and 16 amino acids from the EK1 lead peptide form 78 non-bonded contacts and 9 hydrogen bonds. The Contact Finder tool also confirmed the interactions between key amino acids in the S protein receptor and EK1 peptide. This tool illustrates the extent of association between the receptor's amino acids and the peptide based on the Delta-ACC (Accessible Contact area of the Complex) value, which indicates the degree of interaction between them. Amino acids with a Delta-ACC score exceeding 35 were analyzed, as shown in Appendix 1 in Supplementary File.

**Table 2.** Interactions Between Important Amino Acids in the Syndrome Coronavirus 2 Proteins and the Lead Peptides Based on the Literature Review

Type of interaction	Amino Acids		Refrences
**Values**	**In EK1**	**In S receptor**	
**Hydrogen bond**	Y30	G932	Structural and functional basis for pan-CoV fusion inhibitors against SARS-CoV-2 and its variants with preclinical evaluation (25)
**Hydrogen bond**	E27	Q935	
**Hydrogen bond**	S29	K933	
**Hydrogen bond**	E28	Q926	
**Hydrogen bond**	K18	S943	
**Hydrogen bond**	E15	S943	
**Ionic interaction**		K947	
	In peptide 5	In RdRp	
**Ionic interaction/ water-mediated hydrogen bond**	Arg2	Asp623	Inhibition of the RNA-dependent RNA polymerase of the SARS-CoV-2 by short peptide inhibitors (15)
**Hydrogen bond**		Thr687	
**Ionic interaction**	Asp4	Mg+2	
**Water-mediated hydrogen bonds**		Glu811	
		Asp618	
	In plectacin	In N	
**Conventional Hydrogen Bond**	LYS30	ASN322	In-silico study of peptide-protein interaction of antimicrobial peptides potentially targeting SARS and SARS-CoV-2 nucleocapsid protein (18)
**Hydrophobic**		ALA324	
		PRO323	
**Hydrophobic**	PHE32	PRO323	
**Hydrogen bond; Electrostatic**		ARG321	
**Hydrophobic**	VAL36	PRO323	
**Pi-Donor Hydrogen Bond, Hydrophobic**	TRP8	ALA235	
**Conventional Hydrogen Bond**		TYR283	
		ARG50	
**Conventional Hydrogen Bond**	ASN5	ARG281	
**Pi-Donor Hydrogen Bond**	CYS28	TYR110	
**Hydrophobic**	CYS4	TYR110	
**Hydrogen bond; Electrostatic**	ASP9	ARG41	

Abbreviation: SARS-CoV-2, syndrome coronavirus 2.

**Table 3.** Interactions Between Important Amino Acids in the Protein S Receptor and the EK1 Peptide Based on the PDBsum Tool

Amino Acids in EK1/in S Receptor	Type of Interaction
**Leu1010**	
Asn953	Strong hydrogen bonds
Gln 949	Non-bonded contacts
**Phe1009**	
Gln957	Non-bonded contacts
Asn 953	
Ala 956	
**Val1007**	
Ala 956	Non-bonded contacts
**Thr1008**	
Ala 956	Non-bonded contacts
**Leu1012**	
Gln949	Hydrogen bond
**Asp1011**	
Gln949	Non-bonded contacts
**Leu1036**	
Tyr917	Hydrogen bond
**Leu1019**	
Tyr941	Non-bonded contacts
Ala 942	
Leu945	
**Leu1033**	
Asn925	Non-bonded contacts
**Ser1029**	
Phe927	Non-bonded contacts
**Ile1031**	
Asn928	Strong hydrogen bonds
Phe927	Non-bonded contacts
**Tyr1030**	
Asn928	Non-bonded contacts
Gly932	
Ile931	
Gln935	Hydrogen bond
**Glu1027**	
Gln935	Hydrogen bond
**Leu1026**	
Ile931	Non-bonded contacts
Leu 938	
**Ile1023**	
Leu938	Non-bonded contacts
Ser 939	
Gln935	Hydrogen bond
**Met1016**	
Gly946	Non-bonded contacts

**Figure 3.**
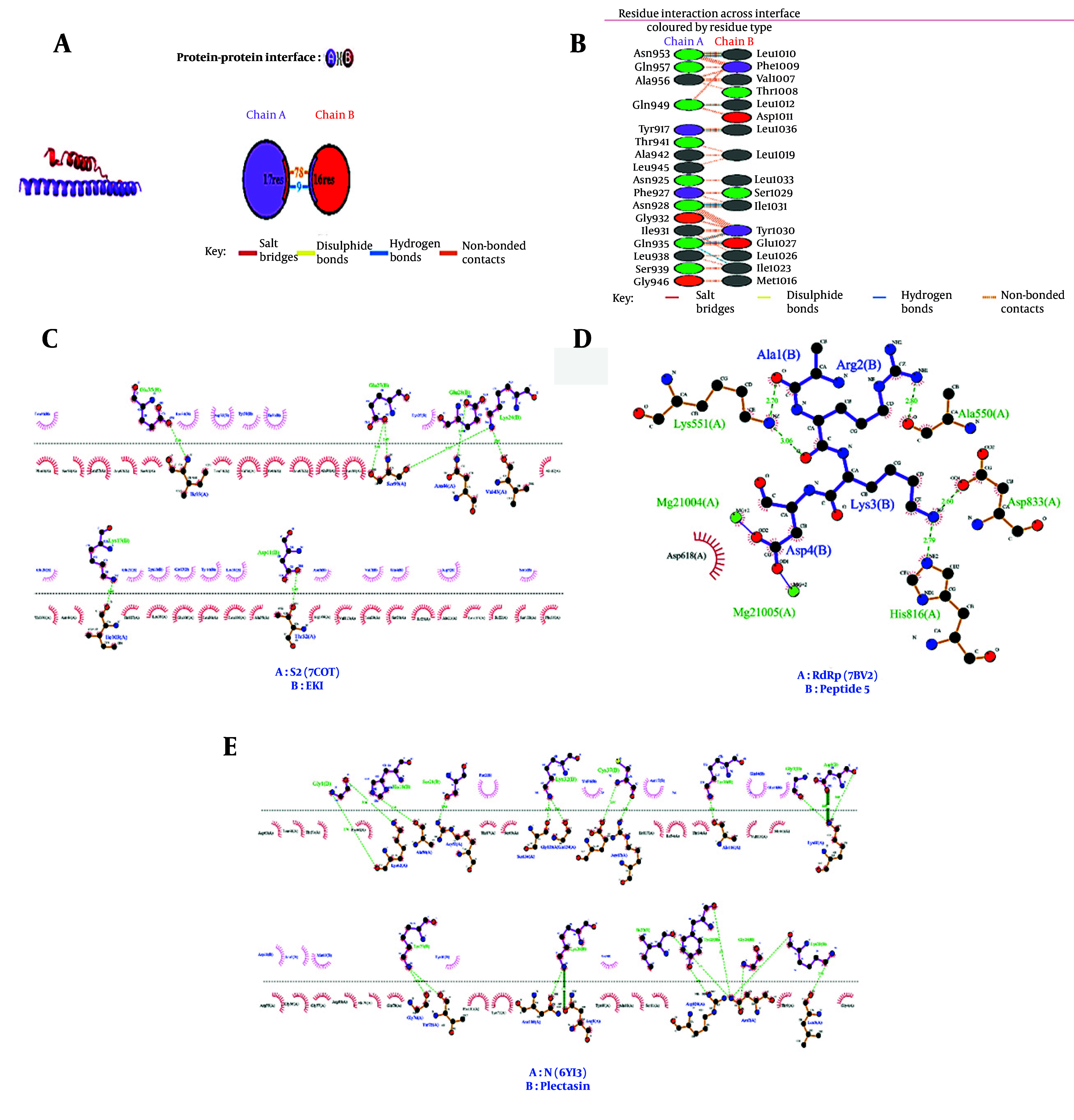
Interactions between amino acids of receptors and lead peptides. A, interactions between the lead peptide (EK1) and the S protein receptor by the PDBsum tool, Chain A represents the receptor, and Chain B represents the lead peptide; B, type and number of bonds between the lead peptide EK1 and the S protein; C, diagrammatic sketch illustrating the interactions between the S receptor (S2 subunit) and EK1; D, interactions between the RdRp receptor and peptide 5; E, interactions between the N receptor and Plectasin. Ligand is shown in purple, and green dashed lines indicate hydrogen bonds with distance in angstroms (Å), spoked red arcs indicate hydrophobic contacts, atoms are shown in black for carbon, blue for nitrogen, red for oxygen, green for fluorine, and yellow for sulfur.

Key amino acid interactions between RdRp and N receptors and their corresponding lead peptides were extracted from a literature survey (Table 2) (15, 18). Subsequently, based on the results obtained from LigPlot software, important amino acid interactions were identified for all three receptors (Figure 3). 

### 4.3. Analogue Design and Energy Minimization

Novel analogues were designed based on a comprehensive set of structural and biochemical principles, including physicochemical properties, helicity, and the key interactions between the inhibitory peptide and the receptor. Essential binding amino acids were identified and conserved within the sequence of the designed analogues. The GRAVY factor, molecular weight (average MW), net charge at neutral pH, isoelectric point, as well as the number of basic, acidic, and neutral amino acids, and hydrophobic amino acids for the lead peptide and its derived analogues were calculated using the Biosynthesis and Novoprolabs tools (Appendix 2 in Supplementary File). A total number of 32 analogues for EK1, 7 analogues for Peptide 5, and 20 analogues for Plectasin were designed. Then, the secondary structure of the lead peptide and the designed analogues was investigated via Secondary Structure Prediction using Chou-Fasman, GOR, and Neural Network tools. Additionally, the antiviral effect of the peptides was assessed using the AVPPred tool (Composition Model and Physicochemical Model) (Table 4). 

**Table 4.** Secondary Structure Analysis, Antiviral Effect Prediction, and Relative Docking Energies of Lead Peptides and Their Analogues against S, RdRp, and N Receptors

No.	Name	Result of Secondary Structure Prediction by Chou-Fasman, GOR and Neural Network	Composition Model%	Physicochemical Model%	Relative Docking Score of ncovDock2 a.u.
**S protein**					
1	EK1	CF: CCCEEEEEHHHHHHHHHHHHHHHHHHHEEECCCCCC; GOR: CETTCEEEEHHHHHHHHHHHHHHHHHHHHHHHHHHH; NN: CCCCHHHHHHHHHHHHHHHHHHHHHHHHHHHHHHHH; Joint:CCCCCEEEHHHHHHHHHHHHHHHHHHHHHHHHHHHH	52.22	63.86	-284.12
2	E	CF: CCCEEEEEHHHHHHHHHHHHHHHHHHHEEECCCCCC; GOR: THHHHEEEHHHHHHHHHHHHHHHHHHHHHHHHHHHH; NN: CCCCEEEHHHHHHHHHHHHHHHHHHHHHHHHHHHHH; Joint:CCCCEEEEHHHHHHHHHHHHHHHHHHHHHHHHHHHH	52.47	64.04	-288.63
3	F	CF: CCEEEEEEHHHHHHHHHHHHHHHHHHHEEECCCCCC; GOR: CCCHHEEEEHHHHHHHHHHHHHHHHHHHHHHHHHHH; NN: CCCCEEEEHHHHHHHHHHHHHHHHHHHHHHHHHHHH; Joint: CCCCEEEEHHHHHHHHHHHHHHHHHHHHHHHHHHHH	53.01	63.98	-289.75
4	G	CF: CCCEEEEEHHHHHHHHHHHHHHHHHHHEEECCCCCC; GOR: THHHHEEEHHHHHHHHHHHHHHHHHHHHHHHHHHHH; NN: CCCCEEEHHHHHHHHHHHHHHHHHHHHHHHHHHHHH; Joint :CCCCEEEEHHHHHHHHHHHHHHHHHHHHHHHHHHHH	50.15	63.91	-288.4
5	H	CF: CEEEEEEEHHHHHHHHHHHHHHHHHHHEEECCCCCC; GOR: CTCHEEEEHHHHHHHHHHHHHHHHHHHHHHHHHHHH; NN: CCCEEEEEEHHHHHHHHHHHHHHHHHHHHHHHHHHH; Joint: CCCEEEEEHHHHHHHHHHHHHHHHHHHHHHHHHHHH	56.94	63.8	-268.3
6	I	CF: CCCEEEEEHHHHHHHHHHHHHHHHHHHEEECCCCCC; GOR: THHHHHEEHHHHHHHHHHHHHHHHHHHHHHHHHHHH; NN: CCCCEEEHHHHHHHHHHHHHHHHHHHHHHHHHHHHH; Joint: CCCCEEEEHHHHHHHHHHHHHHHHHHHHHHHHHHHH	54.01	64.03	-277.95
7	L	CF: CCCEEEEEHHHHHHHHHHHHHHHHHHHEEECCCCCC; GOR: HHHHHHHHHHHHHHHHHHHHHHHHHHHHHHHHHHHH; NN: CCCHHHHHHHHHHHHHHHHHHHHHHHHHHHHHHHHH; Joint: CCCHHHHHHHHHHHHHHHHHHHHHHHHHHHHHHHHH	51.98	64.1	-275.15
8		CF: CCCEEEEEHHHHHHHHHHHHHHHHHHHEEECCCCCC; GOR: HHHHHHEEHHHHHHHHHHHHHHHHHHHHHHHHHHHH; NN: CCCHHHHHHHHHHHHHHHHHHHHHHHHHHHHHHHHH; Joint: CCCCCCEEHHHHHHHHHHHHHHHHHHHHHHHHHHHH	54.01	64.66	-268.6
9	P	CF: CCCEEEEEHHHHHHHHHHHHHHHHHHHEEECCCCCC; GOR: HHHHHHEEHHHHHHHHHHHHHHHHHHHHHHHHHHHH; NN: CCCHHHHHHHHHHHHHHHHHHHHHHHCHCHHHHCCC; Joint: CCCCCCEEHHHHHHHHHHHHHHHHHHHHCHHHHCCC	54.4	64.78	-261.99
10	S	CF: CCCEEEEEHHHHHHHHHHHHHHHHHHHEEECCCCCC; GOR: THHHHHEEHHHHHHHHHHHHHHHHHHHHHHHHHHHH; NN: CCCCEEHHHHHHHHHHHHHHHHHHHHHHHHHHHHHH; Joint: CCCCEEEEHHHHHHHHHHHHHHHHHHHHHHHHHHHH	54.37	64.53	-261.54
11	T	CF: CCCEEEEEHHHHHHHHHHHHHHHHHHHEEECCCCCC; GOR: THHHHHEEHHHHHHHHHHHHHHHHHHHHHHHHHHHH; NN: CCCCEEHHHHHHHHHHHHHHHHHHHHCHCHHHHCCC; Joint: CCCCEEEEHHHHHHHHHHHHHHHHHHHHCHHHHCCC	55.76	64.76	-277.41
12	U	CF: CCCEEEEEHHHHHHHHHHHHHHHHHHHEEECCCCCC; GOR: THHHHHEEHHHHHHHHHHHHHHHHHHHHHHHHHHHH; NN: CCCCEEHHHHHHHHHHHHHHHHHHHHHHHHHHHCCC; Joint: CCCCEEEEHHHHHHHHHHHHHHHHHHHHHHHHHCCC	50.31	64.17	-245.55
13	V	CF: CCCEEEEEHHHHHHHHHHHHHHHHHHHEEECCCCCC; GOR: THHHHHEEHHHHHHHHHHHHHHHHHHHHHHHHHHHH; NN: CCCCEEHHHHHHHHHHHHHHHHHHHHHHHHHHHCCC; Joint: CCCCEEEEHHHHHHHHHHHHHHHHHHHHHHHHHCCC	50.31	64.17	-245.55
14	W	CF: CCCEEEEEHHHHHHHHHHHHHHHHHHHEEECCCCCC; GOR: HHHHHCECHHHHHHHHHHHHHHHHHHHHHHHHHHHH; NN: HHHHHHHHHHHHHHHHHHHHHHHHHHHHHHHHHHHH; Joint: HHHHHCCCHHHHHHHHHHHHHHHHHHHHHHHHHHHH	54.06	63.92	-303.41
15	Y	CF: CCCEEEEEHHHHHHHHHHHHHHHHHHHEEECCCCCC GOR: HHHHHHEEHHHHHHHHHHHHHHHHHHHHHHHHHHHH NN: HHHHHHHHHHHHHHHHHHHHHHHHHHHHHHHHHHHH Joint: HHHHHHEEHHHHHHHHHHHHHHHHHHHHHHHHHHHH	52.33	64.06	-290.33
16	Z	CF: CCCEEEEEHHHHHHHHHHHHHHHHHHHEEECCCCCC; GOR: HHHHHHEEHHHHHHHHHHHHHHHHHHHHHHHHHHHH; NN: HHHHHHHHHHHHHHHHHHHHHHHHHHHHHHHHHHHH Joint: HHHHHHEEHHHHHHHHHHHHHHHHHHHHHHHHHHHH	57.15	64.29	-275.39
17	1	CF: CCCEEEEEHHHHHHHHHHHHHHHHHHHEEECCCCCC; GOR: HHHHHHEEHHHHHHHHHHHHHHHHHHHHHHHHHHHH; NN: HHHHHHHHHHHHHHHHHHHHHHHHHHCHCHHHHCCC; Joint: HHHHHHEEHHHHHHHHHHHHHHHHHHHHCHHHHCCC	59.26	64.41	-300.87
18	2	CF: CCCEEEEEHHHHHHHHHHHHHHHHHHHHCHHHHCCC; GOR: HHHHHHEEHHHHHHHHHHHHHHHHHHHHHHHHHHHH; NN: HHHHHHHHHHHHHHHHHHHHHHHHHHHHHHHHHHHC; Joint: HHHHHHEEHHHHHHHHHHHHHHHHHHHHHHHHHHHC	55.24	64.7	-286.91
19	3	CF: CCCEEEEHHHHHHHHHHHHHHHHHHHHEEECCCCCC; GOR: HHHHHHHHHHHHHHHHHHHHHHHHHHHHHHHHHHHH; NN: HHHHHHHHHHHHHHHHHHHHHHHHHHHHHHHHHHHH; Joint: HHHHHHHHHHHHHHHHHHHHHHHHHHHHHHHHHHHH	52	64.13	-275.17
20	4	CF: CCCEEEEHHHHHHHHHHHHHHHHHHHHEEECCCCCC; GOR: HHHHHHHHHHHHHHHHHHHHHHHHHHHHHHHHHHHH; NN: HHHHHHHHHHHHHHHHHHHHHHHHHHHHHHHHHHHH; Joint: HHHHHHHHHHHHHHHHHHHHHHHHHHHHHHHHHHHH	53.6	64.08	-283.11
21	5	CF: CCCEEEEHHHHHHHHHHHHHHHHHHHHEEECCCCCC; GOR: HHHHHHHHHHHHHHHHHHHHHHHHHHHHHHHHHHHH; NN: HHHHHHHHHHHHHHHHHHHHHHHHHHHHHHHHHHHH; Joint: HHHHHHHHHHHHHHHHHHHHHHHHHHHHHHHHHHHH	60.44	64.06	-292.36
**RdRp receptor**					
1	Peptide 5	ARKD	40.74	19.15	-121.3
2	A1	ARKE	42.01	29.36	-156.64
3	A2	ARHE	41.67	21.44	-117.56
A3	4	HRKE	41.68	40.96	-161.78
A4	5	DRKE	42.15	39.34	-134.93
6	A5	DRRE	42.07	24.44	-187.36
7	A6	ARRE	42.06	18.03	-181.11
8	A7	HRDE	41.87	25.86	-154.6
**N receptor**					
1	Plectasin	CF: CCCCCCHHHHHHHCCCCCCCEECCCCEECCCCEEEEECCC; GOR: EECCCCCCCHHHHHHHTTTTTCTTCTTCCCTTTCCEEETT; NN: CCCCCCCCCCCHHHHHCCCCCCCCCCCCCCCCCCCEEECC; Joint: CCCCCCCCCHHHHHHHCCCCCCCCCCCCCCCCCCCEEECC	48.45	64.09	-255.48
2	A1	CF: CCCCCCHHHHHHHCCCCCCCEECCCCEECCCCEEEEECCC; GOR: EECCTCCCCHHHHHHHTTTTTCTTCTTCCCTTTCCEEETT; NN: CCCCCCCCCCCHHHHHCCCCCCCCCCCCCCCCCCCEEECC; Joint: CCCCCCCCCHHHHHHHCCCCCCCCCCCCCCCCCCCEEECC	50.28	64.13	-250.67
3	A2	CF:CCCCCCHHHHHHHCCCCCCCEECCCCEECCCCEEEEECCC; GOR:ETTCTCCCCHHHHHHHTTTTTCTTCTTCCCTTTCCEEETT; NN:CCCCCCCCCCCHHHHHCCCCCCCCCCCCCCCCCCCEEECC; Joint:CCCCCCCCCHHHHHHHCCCCCCCCCCCCCCCCCCCEEECC	50.36	64.11	-284.75
4	A3	CF: CCCCCCHHHHHHHCCCCCCCEECCCCEECCCCCCCCCCCC; GOR: EECCCCCCCHHHHHHHTTTTTCTTCTTCCCTTTCCHHTTT; NN: CCCCCCCCCCCHHHHHCCCCCCCCCCCCCCCCCCCCCCCC; Joint: CCCCCCCCCHHHHHHHCCCCCCCCCCCCCCCCCCCCCCCC	46.49	64.07	-275.13
5	A4	CF: CCCCCCCHHHHHHCCCCCCCEECCCCEECCCCEEEEECCC; GOR: ETTCCCCCCCTHHHHHTTTTTCTTCTTCCCTTTCCEEETT; NN: CCCCCCCCCCCCHHHHCCCCCCCCCCCCCCCCCCCEEECC; Joint: CCCCCCCCCCCHHHHHCCCCCCCCCCCCCCCCCCCEEECC	48.53	64.09	-300.87
6	A5	CF: CCCCCCHHHHHHHCCCCCCCEECCCCEECCCCCCCCCCCC; GOR: ETTCTCCCCHHHHHHHTTTTTCTTCTTCCCTTTCCHHTTT; NN: CCCCCCCCCCCHHHHHCCCCCCCCCCCCCCCCCCCCCCCC; Joint: CCCCCCCCCHHHHHHHCCCCCCCCCCCCCCCCCCCCCCCC	48.43	64.09	-307.87
7	A6	CF: CCCCCCHHHHHHHCCCCCCCEECCCCEECCCCCCCCCCCC; GOR: ETTCTCCCCHHHHHHHTTTTTCTTCTTCEEETTCCHHTTT; NN: CCCCCCCCCCCHHHHHCCCCCCCCCCCCCCCCCCCCCCCC; Joint: CCCCCCCCCHHHHHHHCCCCCCCCCCCCCCCCCCCCCCCC	47.75	64.10	-276.29
8	A7	CF: CCCCCCHHHHHHHCCCCCCCEECCCCEEECCCCCCCCCCC; GOR: ETTCTCCCCHHHHHHHTTTTTCTTCTTTCEETTTCHHTTT; NN: CCCCCCCCCCCHHHHHCCCCCCCCCCCCCECCCCCCCCCC; Joint: CCCCCCCCCHHHHHHHCCCCCCCCCCCCCCCCCCCCCCCC	47.51	64.10	-317.69
9	A8	CF: CCCCCCHHHHHHHCCCCCCCEECCCCEECCCCEECEECCC; GOR: ETTCTCCCCHHHHHHHTTTTTCTTCTTCEEETTCCEEEET; NN: CCCCCCCCCCCHHHHHCCCCCCCCCCCCCCCCCCCEEEEC; Joint: CCCCCCCCCHHHHHHHCCCCCCCCCCCCCCCCCCCEEEEC	50.25	64.12	-280.84
10	A9	CF: CCCCCCHHHHHHHCCCCCCCEECCCCEEECCCEECEECCC; GOR: ETTCTCCCCHHHHHHHTTTTTCTTCTTCEEETTTCEEEET; NN: CCCCCCCCCCCHHHHHCCCCCCCCCCCCCECCCCCEEECC; Joint: CCCCCCCCCHHHHHHHCCCCCCCCCCCCEECCCCCEEECC	49.94	64.14	-280.24
11	A10	CF: CCCCCCHHHHHHHCCCCCCCEECCCCEEECCCCCCCCCCC; GOR: ETTCTCCCCHHHHHHHTTTTTCTTCTTCEEETTTCHTTTT; NN: CCCCCCCCCCCHHHHHCCCCCCCCCCCCCECCCCCCCCCC; Joint: CCCCCCCCCHHHHHHHCCCCCCCCCCCCEECCCCCCCCCC	47.01	64.11	-281.52
12	A11	CF: EECCCCHHHHHHHCCCCCCCEECCCCEEECCCCCCCCCCC; GOR: TTTCTCCCCHHHHHHHTTTTTCTTCTTTCEETTTCHHTTT; NN: CCCCCCCCCCCHHHHHCCCCCCCCCCCCCECCCCCCCCCC; Joint: CCCCCCCCCHHHHHHHCCCCCCCCCCCCCCCCCCCCCCCC	48.04	64.12	-256.88
13	A12	CF: CCCCCCHHHHHHHHCCCCCCEECCCCEEECCCCCCCCCCC; GOR: ETTCTCCCCTTHHHHHHTTTTCTTCTTTCEETTTCHHTTT; NN: CCCCCCCCCCCHHHHHCCCCCCCCCCCCCECCCCCCCCCC; Joint: CCCCCCCCCCCHHHHHCCCCCCCCCCCCCCCCCCCCCCCC	47.18	64.10	-312.53
14	A13	CF: CCCCCCCHHHHHHCCCCCCCEECCCCEEECCCCCCCCCCC; GOR: ETTCCCCCCCTHHHHHTTTTTCTTCTTTCEETTTCHHTTT; NN: CCCCCCCCCCCCHHHHCCCCCCCCCCCCCECCCCCCCCCC; Joint: CCCCCCCCCCCHHHHHCCCCCCCCCCCCCCCCCCCCCCCC	46.12	64.08	-305.09
15	A14	CF: CCCCCCCHHHHHHCCCCCCCEECCCCEECCCCCCCCCCCC; GOR: ETTCCCCCCCTHHHHHTTTTTCTTCTTCCCTTTCCHHTTT; NN: CCCCCCCCCCCCHHHHCCCCCCCCCCCCCCCCCCCCCCCC; Joint: CCCCCCCCCCCHHHHHCCCCCCCCCCCCCCCCCCCCCCCC	46.74	64.08	-260.52
16	A15	CF: CCCCCCCHHHHHHCCCCCCCEECCCCEEECCCEEEEECCC; GOR: ETTCCCCCCCTHHHHHTTTTTCTTCTTTCEETTTCEEETT; NN: CCCCCCCCCCCCHHHHCCCCCCCCCCCCCECCCCCEEECC; Joint: CCCCCCCCCCCHHHHHCCCCCCCCCCCCCCCCCCCEEECC	48.00	64.10	-260.6
17	A16	CF: CCCCCCHHHHHHHCCCCCCCEECCCCEECCCCEEEEECCC; GOR: EECCCCCCCHTHHHHHTTTTTCTTCTTCCCTTTCCEEETT; NN: CCCCCCCCCCCHHHHHCCCCCCCCCCCCCCCCCCCEEECC; Joint: CCCCCCCCCCCHHHHHCCCCCCCCCCCCCCCCCCCEEECC	48.72	64.11	-293.04
18	A17	CF:CCCCCCHHHHHHHCCCCCCCEECCCCEECCCCEEEEECCC; GOR:EECCCCCCCHHHHHHHTTTTTCTTCTTCCCTTTCCEEETT; NN:CCCCCCCCCCCHHHHHCCCCCCCCCCCCCCCCCCCEEECC; Joint:CCCCCCCCCHHHHHHHCCCCCCCCCCCCCCCCCCCEEECC	47.67	64.11	-253.5
19	A18	CF:CCCCCCHHHHHHHCCCCCCCEECCCCEECCCCEEEEECCC; GOR:TTCCCCCCCHHHHHHHTTTTTCTTCTTCCCTTTCCEEETT; NN:CCCCCCCCCCCHHHHHCCCCCCCCCCCCCCCCCCCEEECC; Joint:CCCCCCCCCHHHHHHHCCCCCCCCCCCCCCCCCCCEEECC	48.49	64.09	-306.04
20	A19	CF: CCCCCCCHHHHHHCCCCCCCEECCCCEECCCCEEEEECCC; GOR: EECCCCCCCCTHHHHHTTTTTCTTCTTCCCTTTCCEEETT; NN: CCCCCCCCCCCCHHHHCCCCCCCCCCCCCCCCCCCEEECC; Joint: CCCCCCCCCCCHHHHHCCCCCCCCCCCCCCCCCCCEEECC	48.40	64.10	-267.38
21	A20	CF: CCCCCCHHHHHHHCCCCCCCEECCCCEECCCCEEEEECCC; GOR: EECCCCCCCHHHHHHHTTTTTCTTCTTCCCTTTCCEEETT; NN: CCCCCCCCCCCHHHHHCCCCCCCCCCCCCCCCCCCEEECC; Joint: CCCCCCCCCHHHHHHHCCCCCCCCCCCCCCCCCCCEEECC	48.43	64.10	-307.01

Following the design process, the three-dimensional structures of the lead peptides and their derived analogues were modeled utilizing the AlphaFold2 tool. To ensure the accuracy of the predicted models and to achieve stable states, energy minimization of all structures was performed using the Amber force field.

The overall quality of the predicted three-dimensional structural models was assessed and validated using the predicted local distance difference test (pLDDT) confidence metric. For the analogues designed to target the S, N, and RdRp proteins, all models consistently achieved average pLDDT scores ranging from 59.5 to 89.8. This high confidence range indicates that the predicted backbone structures are highly reliable and suitable for subsequent molecular docking analyses.

**Figure 4.**
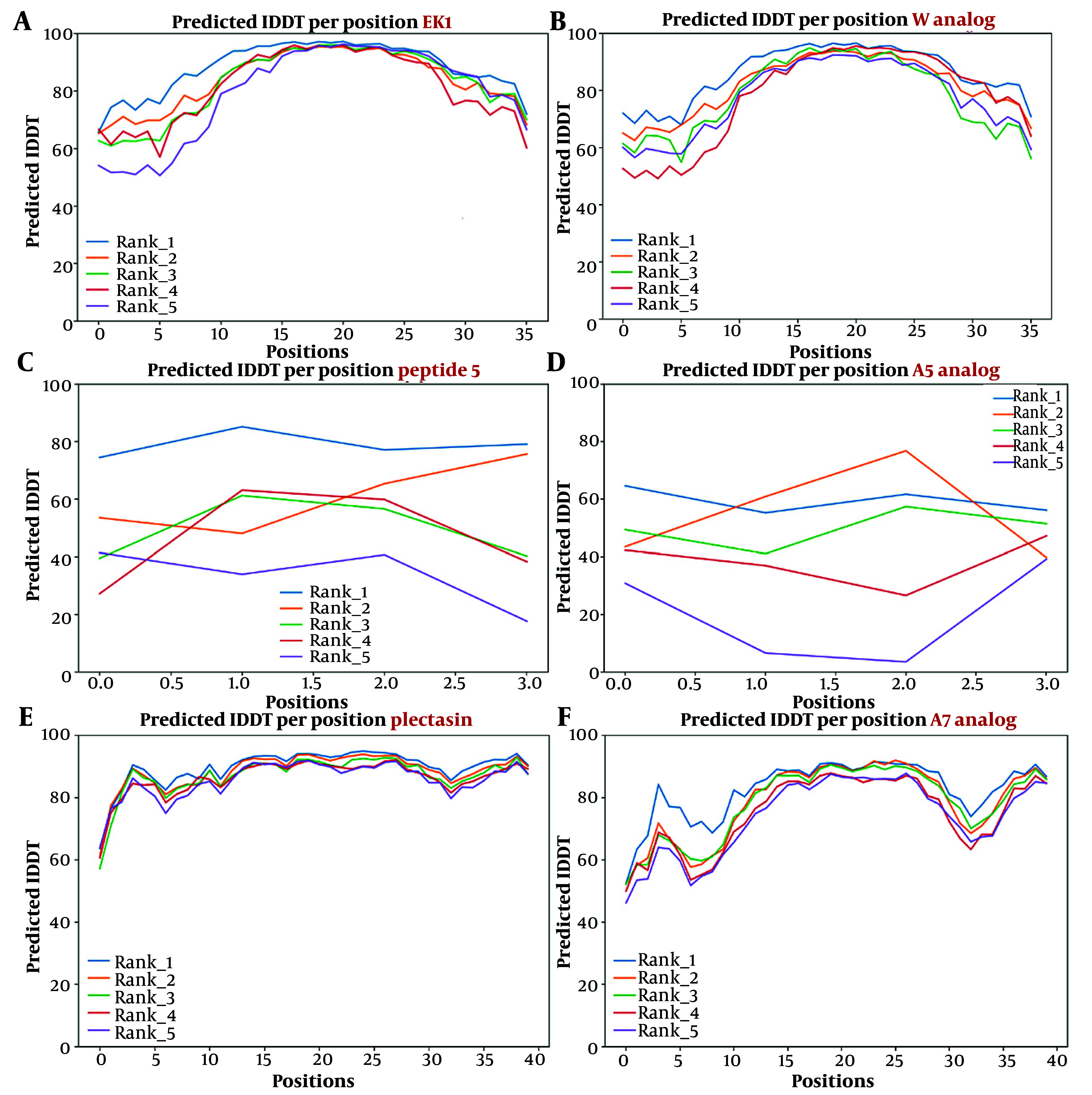
Predicted local distance difference test (pLDDT) plots for lead peptides and designed analogues. These plots illustrate the confidence level of the predicted 3D structure (the peptide backbone) at each amino acid position. Higher lDDT scores (closer to 100) indicate higher quality and reliability of the modeling. Each colored line (rank) represents a different predicted model. A, plot for the EK1 lead peptide; B, plot for the EK1 analogue W; C, plot for the peptide 5 lead peptide; D, plot for the peptide 5 analogue A7; E, plot for the Plectasin lead peptide; F, plot for the Plectasin analogue A5.

For each target, the lead peptide and the top-scoring analogue were selected to represent the quality of the entire dataset. Specifically, the lead peptide for the S protein obtained an average pLDDT score of 88.2, while its analogue with the best relative docking score scored 85.9. Similarly, the lead peptide for the N protein and its best relative docking score analogue had average pLDDT scores of 89.8 and 89.7, respectively. Finally, the lead and top relative docking score analogue for the RdRp protein achieved average scores of 79.1 and 59.5. These results collectively confirm the high structural quality and reliability of the designed peptide models, providing a solid foundation for the reported docking results. The corresponding pLDDT plots for these selected peptides are presented in Figure 4. 

Moreover, the toxicity of the designed peptide analogues was assessed via the ToxinPred tool. The SVM models that utilize various amino acid sequence features such as amino acid composition, dipeptide composition, motif presence, and quantitative matrix (QM) models were employed in this module to create a foundation for analyzing and designing peptides with specific toxicity (26). The data obtained from this tool are listed in Table 5. 

**Table 5.** Toxicity Assessment of Lead and Designed Analogue Peptides Targeting Viral Proteins S, RdRp, and N

No	Name	Toxicity Status	SVM Score	Sequence
**Inhibitory peptides of S protein**				
1	EK1	Non-Toxic	-1.10	SLDQINVTFLDLEYEMKKLEEAIKKLEESYIDLKEL
2	E	Non-Toxic	-1.00	SLDEIIVTFLDLEYEMKKLEEAIKKLEESYIDLKEL
3	F	Non-Toxic	-1.05	SLDDIIVTFLDLEYEMKKLEEAIKKLEESYIDLKEL
4	G	Non-Toxic	-0.86	SADEIIVTFLDLEYEMKKLEEAIKKLEESYIDLKEL
5	H	Non-Toxic	-0.91	SADYIIVTFLDLEAEMKKLEEAIKKLEESYIDLKEL
6	I	Non-Toxic	-0.99	SADEIIVTFLDLEAEMKKLEEAIKKLEESYIDLKEL
7	L	Non-Toxic	-1.01	EADEIIVTFLDLELEMKKLEEAIKKLEESYIDLKEL
8	O	Non-Toxic	-0.83	QADEIIVTFLDLELEMKKLEEAIKKLEESYIDLREL
9	P	Non-Toxic	-0.96	QADEIIVTFLDLELEMKKLEEAIKKLEESYIDLRDL
10	S	Non-Toxic	-0.86	SADEIIVTFLDLELEMKKLEEAIKKLEESYIDLREL
11	T	Non-Toxic	-0.98	SADEIIVTFLDLELEMKKLEEAIKKLEESYIDLRDL
12	U	Non-Toxic	-1.05	SADEIIVTFLDLELEMKKLEEAIKKLEESYIDLKDL
13	V	Non-Toxic	-1.05	SADEIIVTFLDLELEMKKLEEAIKKLEESYIDLKDL
14	W	Non-Toxic	-1.01	ALAQINVTFLDLEYEMKKLEEAIKKLEESYIDLKEL
15	Y	Non-Toxic	-0.93	ALAEIIVTFLDLEYEMKKLEEAIKKLEESYIDLKEL
16	Z	Non-Toxic	-0.73	ALAEIIVTFLDLEYEMKKLEEAIKKLEESYIDLREL
17	1	Non-Toxic	-0.88	ALAEIIVTFLDLEYEMKKLEEAIKKLEESYIDLRDL
18	2	Non-Toxic	-1.10	ALAEIIVTFLDLEYEMKKLEEAIKKLEESYLDLRDL
19	3	Non-Toxic	-0.89	ALAEINVQFLDLEYEMKKLEEAIKKLEESYIDLKEL
20	4	Non-Toxic	-0.78	ALAEIIVQFLDLEYEMKKLEEAIKKLEESYIDLKEL
21	5	Non-Toxic	-0.75	ALAEIIVQFLDLEYEMKKLEEAIKKLEESYIDLKAL
22	A	Non-Toxic	-1.11	SLDEINVTFLDLEYEMKKLEEAIKKLEESYIDLKEL
23	B	Non-Toxic	-1.15	SLDDINVTFLDLEYEMKKLEEAIKKLEESYIDLKEL
24	C	Non-Toxic	-1.02	SLDYINVTFLDLEYEMKKLEEAIKKLEESYIDLKEL
25	D	Non-Toxic	-1.00	SLDQIIVTFLDLEYEMKKLEEAIKKLEESYIDLKEL
26	J	Non-Toxic	-1.03	SADEIIVTFLDLELEMKKLEEAIKKLEESYIDLKEL
27	K	Non-Toxic	-1.03	SADEIIVTFLDLELEMKKLEEAIKKLEESYIDLKEL
28	M	Non-Toxic	-1.00	QADEIIVTFLDLELEMKKLEEAIKKLEESYIDLKEL
29	N	Non-Toxic	-1.00	QADEIIVTFLDLELEMKKLEEAIKKLEESYIDLKEL
30	Q	Non-Toxic	-1.02	QADEIIVTFLDLELEMKKLEEAIKKLEESYIDLKDL
31	R	Non-Toxic	-1.02	QADEIIVTFLDLELEMKKLEEAIKKLEESYIDLKDL
32	X	Non-Toxic	-1.04	ALAEINVTFLDLEYEMKKLEEAIKKLEESYIDLKEL
33	6	Non-Toxic	-0.93	SLDYIIVTFLDLEYEMKKLEEAIKKLEESYIDLKEL
**Inhibitory peptides of RdRp protein**				
1	peptide 5	Non-Toxic	-0.89	ARKD
2	A1	Non-Toxic	-0.83	ARKE
3	A2	Non-Toxic	-0.83	ARHE
4	A3	Non-Toxic	-0.70	HRKE
5	A4	Non-Toxic	-0.82	DRKE
6	A5	Non-Toxic	-0.85	DRRE
7	A6	Non-Toxic	-0.87	ARRE
8	A7	Non-Toxic	-0.71	HRDE
**Inhibitory peptides of N protein**				
1	Plectasin	Toxic	0.85	GFGCNGPWDEDDMQCHNHCKSIKGYKGGYCAKGGFVCKCY
2	A1	Toxic	0.81	GFGCNGPWEEDDMQCHNHCKSIKGYKGGYCAKGGFVCKCY
3	A2	Toxic	0.68	GFGCNGPYEEDDMQCHNHCKSIKGYKGGYCAKGGFVCKCY
4	A3	Toxic	0.89	GFGCNGPWDEDDMQCHNHCKSIKGYKGGYCAKGGFACKCY
5	A4	Toxic	0.74	GFGCNGPYDEDDMQCHNHCKSIKGYKGGYCAKGGFVCKCY
6	A5	Toxic	0.72	GFGCNGPYEEDDMQCHNHCKSIKGYKGGYCAKGGFACKCY
7	A6	Toxic	0.76	GFGCNGPYEEDDMQCHNHCKSIKGYKGGYCAKGGWACKCY
8	A7	Toxic	0.56	GFGCNGPYEEDDMQCHNHCKSIKGYKGGYCARGGFACKCY
9	A8	Toxic	0.66	GFGCNGPYEEDDMQCHNHCKSIKGYKGGYCAKGGAVCKCY
10	A9	Toxic	0.49	GFGCNGPYEEDDMQCHNHCKSIKGYKGGYCARGGAVCKCY
11	A10	Toxic	0.60	GFGCNGPYEEDDMQCHNHCKSIKGYKGGYCARGGWACKCY
12	A11	Toxic	0.42	GFGCRGPYEEDDMQCHNHCKSIKGYKGGYCARGGFACKCY
13	A12	Toxic	0.84	GFGCNGPYEEDDMECHNHCKSIKGYKGGYCARGGFACKCY
14	A13	Toxic	0.63	GFGCNGPYDEDDMQCHNHCKSIKGYKGGYCARGGFACKCY
15	A14	Toxic	0.78	GFGCNGPYDEDDMQCHNHCKSIKGYKGGYCAKGGFACKCY
16	A15	Toxic	0.57	GFGCNGPYDEDDMQCHNHCKSIKGYKGGYCARGGFVCKCY
17	A16	Toxic	0.55	GFGCNGPRDEDDMQCHNHCKSIKGYKGGYCAKGGFVCKCY
18	A17	Toxic	0.67	GFGCNGPEDEDDMQCHNHCKSIKGYKGGYCAKGGFVCKCY
19	A18	Toxic	0.61	GFGCNGPFDEDDMQCHNHCKSIKGYKGGYCAKGGFVCKCY
20	A19	Toxic	0.81	GFGCNGPPDEDDMQCHNHCKSIKGYKGGYCAKGGFVCKCY
21	A20	Toxic	0.60	GFGCNGPLDEDDMQCHNHCKSIKGYKGGYCAKGGFVCKCY

### 4.4. Molecular Docking and Interaction Study of Designed Analogues

A relative docking score, in arbitrary units (a.u.), was calculated using the nCoVDock2 tool results for lead peptides and analogues designed for receptors, which are specified in Table 4. In the case of lead peptide 5 and its derived analogues, Arg A2 in the analogues and lead peptides and Asp A623 in the RdRp receptor were considered as constraint options in all cases of docking (pinned-form docking). For the N protein, ASP A9 in the analogues, lead peptide, and ARG A48 in the N receptor were considered as constraint options in all cases of docking.

**Figure 5.**
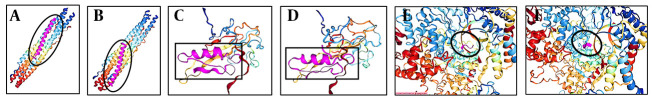
The docking results of the nCoVDock2 tool for lead peptides and the designed analogues with their respective receptors. A, EK1-S receptor complex. EK1 binds to the second HR1 in the S2 subunit of the S protein; B, W analogue-S receptor complex; C, plectasin-N receptor complex. This ligand interacts with chains A and C of the SARS-CoV-2 nucleocapsid via disulfide bonds; D, A7 analogue-N receptor complex; E, peptide 5-RdRp complex. Peptide 5 binds to the nucleotide triphosphate (NTP) insertion sites within the RdRp protein; F, A5 analogue-RdRp complex. Inhibitory peptides are shown in purple.

**Figure 6.**
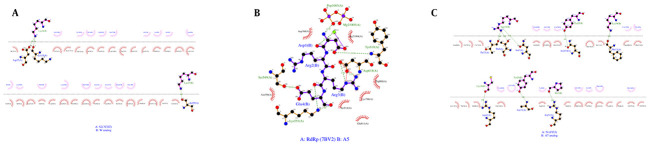
Two-dimensional LigPlot analysis of key protein-analogue peptide interactions. The figure presents the non-covalent interactions for: A, S2 (7COT) and W analogue complex; B, A5 analogue and RdRp (PDB:7BV2) complex; and C, A7 analogue and N protein (PDB:6YI3). These diagrams highlight the crucial role of both hydrogen bonding and hydrophobic interactions in stabilizing the complexes. In the W analogue-S receptor complex, hydrogen bonds between the Lys34 and Asp27, Lys24, and between Lys17 and Ser99 residues are essential for stability. The A5 analogue forms seven hydrogen bonds with RdRp, primarily through its Asp1, Arg3, and Glu4 residues. Similarly, multiple hydrogen bonds also contribute significantly to the stability of the A7 analogue-N protein complex. The hydrophobic interactions are prominent, particularly in the W analogue and A7 analogue complexes, reflecting the design goal to enhance this type of non-covalent binding. (The diagrams follow the standard LigPlot conventions: dotted lines represent hydrogen bonds (with numbers indicating distance in Å), half-circles with spokes indicate hydrophobic contacts, black dots represent the corresponding atoms involved. Chain A is the receptor protein, and chain B is the analogue peptide.)

The images obtained from the molecular docking for the lead peptides and the best analogues derived from them in terms of lowest molecular docking energy are shown in Figure 5. W (ALAQINVT-FLDLEYEMKKLEEAIKKLEESYIDLKEL), A5 (DRRE), and A7 (GFGCNGPYEEDDMQCHNHCKSIKGYKGGYCARG-GFACKCY) analogues were selected as the best analogues for inhibitory interaction with S, RdRp, and N proteins, respectively. Furthermore, the results of the LigPlot analysis revealed interactions between the three best analogues and their respective receptors (Figure 6A-C). It was noticeable that W-S and A7-N receptor complexes formed more hydrophobic contacts than EK1-S and Plectasin-N peptides. Also, the A5-RdRp complex exhibited more hydrogen bond interactions with the amino acid residues ASP 618, Tyr 619, Ser 549, and Lys 551.

### 4.5. Molecular Dynamics

**Figure 7.**
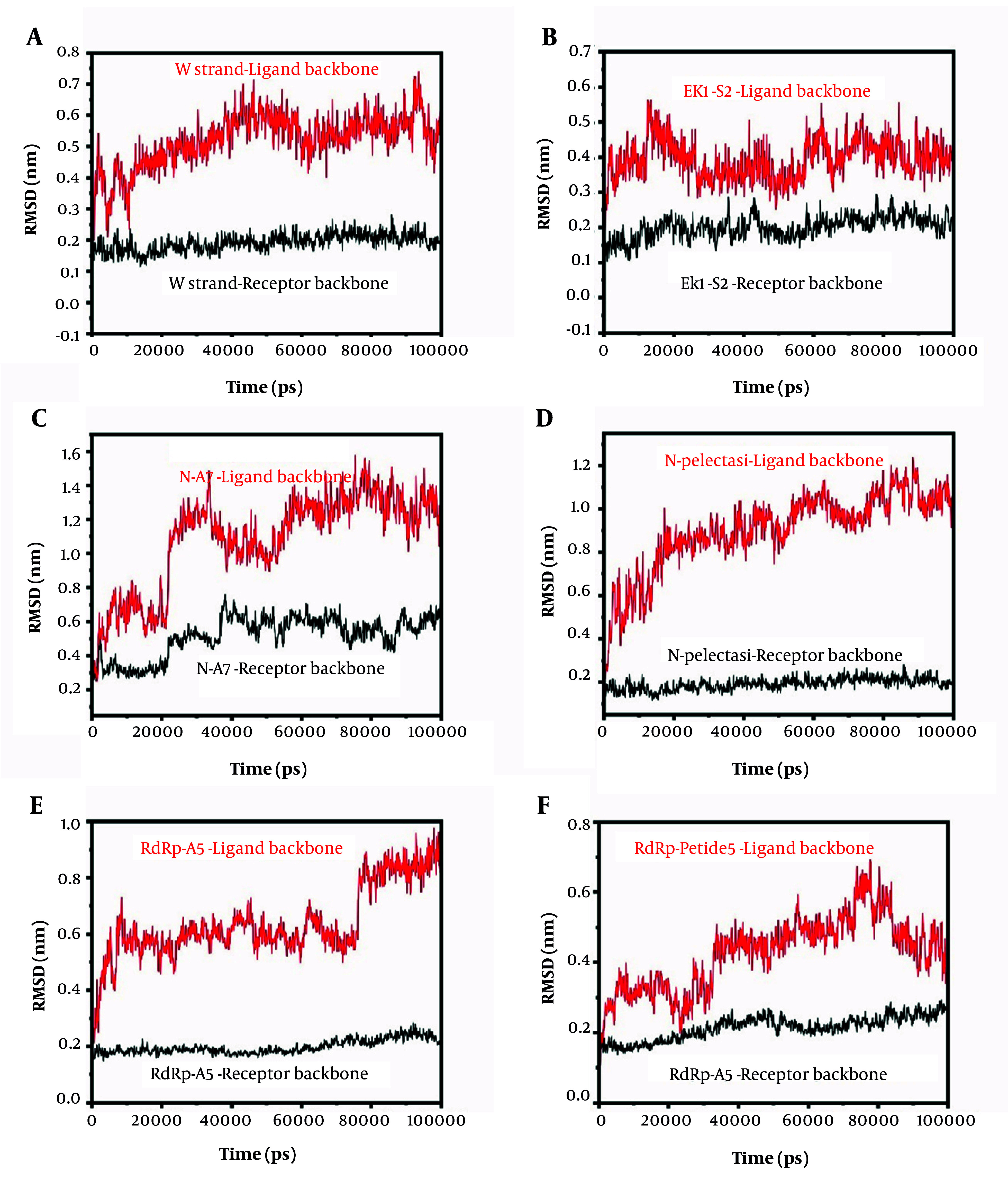
Root mean square deviation (RMSD) trajectories of ligand and receptor backbones for peptide-protein complexes over 100 ns molecular dynamics (MD) simulations. A, W strand ligand (S protein) and receptor backbones; B, EK1-S2 ligand (S protein) and receptor backbones; C, N-A7 ligand (N protein) and receptor backbones; D, N-Plectasin ligand (N protein) and receptor backbones; E, RdRp-A5 ligand (RdRp) and receptor backbones; F, RdRp-Peptide 5 ligand (RdRp) and receptor backbones. RMSD (nm) is plotted against simulation time (ps). Ligand backbones are shown in solid lines, and receptor backbones are shown in dashed lines. All simulations were performed in triplicate.

The structural dynamics and binding stability of the designed peptide-receptor complexes were evaluated via 100 ns MD simulations, with root mean square deviation (RMSD) analyses summarized in Figure 7. Key findings are categorized by target protein below.

#### 4.5.1. Spike (S) Protein Complexes

The W strand ligand backbone exhibited an initial RMSD rise to ~0.8 nm within 40 ns, stabilizing at 0.6 - 0.8 nm thereafter (Figure 7A), indicating moderate conformational flexibility during binding equilibration. The S protein receptor backbone remained stable (RMSD < 0.3 nm), reflecting a rigid binding scaffold. Compared to the EK1 lead peptide (relative docking score: -284.12 a.u.), the W strand's sustained stability suggests improved adaptive interactions.

The EK1-S2 ligand backbone stabilized at 0.5 - 0.7 nm after an initial rise, while the receptor backbone maintained exceptional rigidity (RMSD ~0.1 - 0.3 nm) (Figure 7B). This intermediate flexibility may facilitate adaptive binding without destabilizing the receptor, contrasting with the higher mobility of the W strand.

#### 4.5.2. Nucleocapsid (N) Protein Complexes

The A7-N ligand backbone displayed pronounced flexibility (RMSD peak: ~1.6 nm), stabilizing at 1.2 - 1.4 nm, whereas the N protein receptor remained stable (RMSD ~0.4 - 0.6 nm) (Figure 7C). This divergence highlights ligand-dominated dynamics, potentially reflecting transient interactions.

In the N-Plectasin Complex (Figure 7D), the ligand backbone exhibited similar flexibility (RMSD: 1.2–1.4 nm), while the receptor backbone maintained low fluctuations (RMSD ~0.3 - 0.5 nm). Compared to the Plectasin lead (relative docking score: -255.48 a.u.), the engineered N-Plectasin retained comparable mobility, suggesting retained binding adaptability.

#### 4.5.3. RNA-Dependent RNA Polymerase (RdRp) Complexes

The A5 ligand backbone stabilized at 0.8 - 1.0 nm after an initial rise, outperforming lead peptide 5 (relative docking score: -121.3 a.u.) in stability (Figure 7E). The RdRp receptor backbone showed minimal deviation (RMSD ~0.3 - 0.5 nm), consistent with its conserved enzymatic role.

Peptide 5 (Figure 7F) also achieved greater stability (RMSD: 0.6 - 0.8 nm), comparable to A5. Both receptor backbones remained rigid (RMSD < 0.4 nm).

The simulations reveal a spectrum of stability among the designed peptides, with RdRp-A5 and W strand demonstrating optimal balance between flexibility and receptor engagement. Excessive ligand mobility (e.g., N-A7) may compromise efficacy, warranting structural refinements such as rigidifying mutations or increasing hydrophobic interactions. Conversely, moderate flexibility (e.g., EK1-S2, RdRp-A5) likely facilitates adaptive binding. These findings align with relative docking scores and underscore the importance of dynamic stability in peptide therapeutics. Future work should integrate residue-specific interaction analyses (e.g., hydrogen bond occupancy, MM-PBSA) and experimental validation to refine lead candidates.

## 5. Discussion

Computational drug design has revolutionized pharmaceutical and clinical research by providing rapid, cost-effective methodologies for therapeutic agent development. This paradigm significantly reduces both temporal and financial investments while enabling a comprehensive evaluation of drug efficacy across diverse physiological conditions and structural mutations in the target molecular receptor (27). As a computational tool, molecular docking plays a vital role in understanding the ligand-receptor interactions and determining the binding affinity of drug-like molecules with the receptor. This method is employed to discover new drugs targeting specific research interests. During molecular docking, the molecule binds to various receptor sites. It elucidates the binding affinity regarding energy, aiding in screening the best receptor sites and molecules against targeted receptors based on their binding affinity (28, 29).

The complexity of SARS-CoV-2, characterized by extensive structural mutations and strain diversity, presents unprecedented challenges for traditional drug discovery approaches. Specialized computational platforms, such as nCoVDock2, address these challenges by predicting binding interactions between wild-type and mutant viral targets with various ligand classes, including small molecules, peptides, and antibodies (21, 30). This study leveraged nCoVDock2's global and site-specific docking capabilities to design targeted antiviral peptides against three critical SARS-CoV-2 proteins: Spike (S), nucleocapsid (N), and RNA-dependent RNA polymerase (RdRp).

### 5.1. Spike Protein Targeting and Peptide Design Strategy

The SARS-CoV-2 spike protein orchestrates viral entry through a sophisticated two-step mechanism. The S1 subunit mediates initial receptor recognition via human angiotensin-converting enzyme 2 (hACE2), while the S2 subunit executes membrane fusion through conformational rearrangements. Targeting S1 via hACE2-derived peptides has been investigated in silico and in vitro. Results of this study indicate that targeting S protein via peptides with multiple hydrogen bond interactions can be an effective method for inhibiting SARS-CoV-2 (31). In the S2 subunit that we targeted in our study, heptad repeat regions HR1 and HR2 undergo antiparallel association to form a thermodynamically stable six-helix bundle (6-HB), facilitating viral-cellular membrane proximity essential for fusion.

The HR1-HR2 interaction interface is predominantly hydrophobic, with HR1 trimers creating deep grooves that accommodate hydrophobic residues from HR2 (V1164, L1166, I1169, I1172, A1174, V1176). Critically, the HR1 region exhibits remarkable conservation compared to the hypervariable receptor-binding domain, establishing it as an attractive target for broad-spectrum antiviral development.

Building upon the established efficacy of EK1 — a pan-coronavirus fusion inhibitor derived from HCoV OC43 HR2 that targets the conserved HR1 — we implemented a structure-guided design approach to enhance inhibitory potency. EK1 shows broad and potent inhibitory activity against SARS-CoV-2 infection and its mutants, as well as all the tested HCoVs and some SARS-CoVs, targeting them, leading to inhibition of infection (23-25). Our rational design strategy for EK1 analogues focused on preserving critical residues while introducing mutations to enhance alpha-helical content and hydrophobic-hydrophobic interactions, both of which are crucial for inhibitory efficacy and 6-HB formation. A 2023 study by Quagliata et al. noted that the alpha-helix structure's stability during receptor interaction has a positive impact, and the formation of 6-HB requires the interaction of multiple domains with the alpha-helix structure (32). Determining the secondary structure in lead peptide EK1 to design peptides with higher helicity highlights the importance of increasing helicity in enhancing the binding affinity of EK1 analogues to the S protein receptor and inhibiting it, as indicated. In this research, the basis for generating mutations was to enhance the interaction between the ligand and the analogue by leveraging the increased helicity of these analogues. The mutations in these analogues, which increased helicity according to Table 4, were expected to enhance the docking results, which was successfully achieved. Moreover, the design strategy used amino acids with similar physicochemical properties, such as charge, for substitution. In a study, Yan and Gao aimed to enhance the binding affinity of the EK1 peptide by introducing mutations in its sequence. Their results exhibited an improvement in the relative docking score of the designed analogues (24), which aligns with our strategy and findings.

Computational analysis revealed significant improvements in binding affinity across designed analogues. According to Table 3, notably, analogues W, Y, 1, 2, and 5 demonstrated relative docking scores of -303.41, -290.33, -300.87, -286.91, and -292.36 a.u., respectively, substantially exceeding the parent EK1 peptide (-284.12 a.u.) and control variants E, F, and G (-288.63, -289.75, -288.4 a.u.). Analogue W emerged as the lead candidate with optimal binding characteristics.

Secondary structure predictions using multiple algorithms (Chou-Fasman, Garnier-Osguthorpe-Robson, Neural Network) consistently indicated enhanced helical content in designed analogues relative to EK1. Importantly, physicochemical analysis (Appendix 2 in Supplementary File) confirmed conservation of charge properties at physiological pH, indicating minimal changes in physicochemical properties. This underscores the focus on enhancing structural helicity in the design of these peptides and validates our design strategy's selectivity for structural rather than chemical modifications.

### 5.2. RdRp Inhibitory Peptide Development

Research on RdRp inhibitory peptides has established that several short ionic peptides play a role in inhibiting the RNA binding site in the RdRp receptor. Crystallographic analysis reveals an RNA-binding cavity positioned at the interface of finger, palm, and thumb domains, characterized by a complex electrostatic landscape comprising positively charged residues (Lys545, Lys551, Arg553, Arg555, Lys593, Arg621, Arg631) and negatively charged counterparts (Asp618, Asp623, Asp760, Asp761). These residues are crucial for positioning RNA during replication, RNA strand elongation, and catalysis. The two magnesium ions in the binding site are also likely to have a significant role in catalysis. Additionally, the involvement of some of these amino acids in the insertion of new nucleotides into the RNA strand, referred to as nucleotide triphosphate (NTP) insertion sites, has been suggested. It was also found that these inhibitors can occupy nucleotide triphosphate (NTP) insertion sites, thereby preventing viral genome replication by inhibiting the synthesis of new nucleotides. According to a study by Pant et al. in 2021, it is imperative to design inhibitors that can not only immediately block virus replication by interfering with RdRp function but also produce more sustained effects in humans.

Our approach focused on developing short, ionic peptides capable of disrupting nucleotide triphosphate (NTP) binding sites, thereby preventing viral genome replication. Lead peptide 5, characterized as a tetraionic inhibitor, offered several advantages: minimal toxicity profiles, high target specificity, synthetic accessibility, and resistance to proteolytic degradation due to its compact architecture.

Additionally, similar to nucleotide drugs, this inhibitor is not classified as replication errors, thus minimizing the likelihood of antiviral resistance developing against these peptide drugs. Consequently, this peptide holds promise as a potent candidate for developing additional analogues to target the RdRp receptor (15). The physicochemical properties and charge of amino acids are crucial in the design of analogues for lead peptide 5, as per Appendix 3 in Supplementary File. The focus has been on strengthening the bonds between the amino acids of the analogues and the receptor due to the short length of this lead peptide. Arg2 in lead peptide 5 has a positive charge, while Asp623 in the RdRp receptor has a negative charge, resulting in the formation of a strong electrostatic bond. Thus, Arg2 remains unchanged in the analogue design. Furthermore, Asp4 of lead peptide 5 forms a robust ionic bond with the Mg^2+^ ion of the RdRp receptor, and to enhance this bond, a Glu amino acid with a negative charge was utilized in this position, as per the mentioned table (Table 5). 

Systematic optimization yielded analogues A1, A3, A4, A5, A6, and A7 with substantially improved relative docking scores (-156.64, -161.78, -134.93, -187.36, -181.11, -154.6 a.u.) compared to parent peptide 5 (-121.3 a.u.). Analogue A5 achieved optimal performance (-187.36 a.u.), representing a 68% improvement in binding affinity. Hydropathy analysis (GRAVY values) confirmed the hydrophilic character of designed analogues (Appendix 3 in Supplementary File), consistent with their intended mechanism of aqueous-phase RNA binding site occupation. That aligns with the design logic aimed at creating inhibitory peptides from lead peptide 5 while maintaining and enhancing their relative docking score without altering their main physicochemical properties.

### 5.3. Nucleocapsid Protein Targeting Strategy

The SARS-CoV-2 nucleocapsid (N) protein plays multifaceted roles in viral pathogenesis, including genomic RNA packaging, virion assembly, and host immune evasion. The N-terminal domain (NTD) exhibits distinctive electrostatic properties that facilitate specific interactions with the 3′-terminal region of viral genomic RNA through predominantly electrostatic mechanisms (18, 21). This specificity, coupled with high conservation across coronavirus species, positions the N protein as an attractive therapeutic target.

The selected approach for creating inhibitory peptide analogues for this receptor has focused on enhancing interactions between peptide and target by creating mutations in amino acids that strongly interact with the receptor. The helical structure plays a significant role in these interactions (33). Therefore, mutations were created proportional to the increase in helicity. A hydrophobic amino acid is also positioned adjacent to the specific residues in the receptor to facilitate hydrophobic-hydrophobic interactions. Also, the charge of amino acids is crucial for strengthening the interactions. Moreover, to enhance the affinity, the amino acids in the analogues were altered based on the charge of the original peptide.

We employed Plectasin (a 40-residue antimicrobial defensin with established structural stability) as a scaffold for N protein-targeting peptides. This peptide contains 6 cysteine amino acids that establish 3 disulfide linkages in the folded protein molecule between Cys1 and Cys5, Cys2 and Cys4, and Cys3 and Cys6, thereby stabilizing the α-helix-β-structural motif (34).

Analogues A2, A3, A4, A5, A6, A7, A8, A9, A10, A11, A12, A13, A14, A15, A16, A18, A19, and A20 (Table 2) demonstrated relative docking scores of -284.75, -275.13, -300.87, -307.87, -276.29, -317.69, -280.84, -280.24, -281.52, -256.88, -312.53, -305.09, -260.52, -260.6, -293.04, -306.04, -267.38, and -307.01 a.u., respectively. These scores compared favorably to the Plectasin lead peptide relative docking score of -255.48 a.u., revealing improved and more stable molecular docking results for all analogues. Among 18 designed analogues (A2-A20), variant A7 demonstrated superior binding characteristics with a relative docking score of -317.69 a.u., representing a 24% improvement over parent Plectasin (-255.48 a.u.). This enhancement correlated with optimized hydrogen bonding networks and hydrophobic contacts at the peptide-protein interface. Asp9 of the lead peptide forms electrostatic bonds with Arg41 of the N protein receptor. Notably, analogues modified this interaction by replacing Asp9 with Glu. The importance of Trp8 in establishing hydrophobic interactions was highlighted, with Tyr substitution at this position. Moreover, in row number 3 of Table 2, Val36 formed hydrophobic bonds with Pro323, and a Val-to-Ala mutation in this region improved relative docking scores and enhanced binding stability. Another significant modification involved replacing Lys32 (positive charge) with Arg (also positively charged). According to Appendix 4, the GRAVY factor of the analogues ranged between -1 and zero, with charges at pH 7 relatively similar to the lead peptide Plectasin. These findings emphasize the importance of designing mutations that enhance analogue-receptor affinity compared to lead peptide Plectasin while maintaining physicochemical properties.

### 5.4. Interaction Analysis and Binding Characterization

LigPlot analysis of interactions between designed analogues W, A5, and A7 and target proteins revealed that selected molecules formed strong hydrophobic interactions with polar residues, indicating robust interactions and analogue selectivity toward targets (35, 36). All three designed analogues demonstrated multiple hydrogen bonds with target proteins, crucial for strong ligand-target protein interactions. The obtained interaction profiles align with established principles of high-affinity protein-protein interactions, where binding energy derives from multiple cooperative contacts rather than single high-energy interactions.

For RdRp inhibitors specifically, hydrogen bond multiplicity correlates strongly with inhibitory potency, supporting our design emphasis on polar contact optimization (15, 37). Overall, increased helicity and hydrophobic interactions in designed analogues simultaneously enhance binding strength between analogues and target proteins (33).

### 5.5. Toxicity Assessment and Safety Considerations

Therapeutic peptide development requires comprehensive safety evaluation, as peptides can exhibit cytotoxicity, immunogenicity, or hemolytic activity despite their generally favorable safety profiles. Therefore, evaluating designed drug peptide toxicity is essential, with computational methods for peptide toxicity prediction being developed (38).

ToxinPred represents one such tool that assesses peptide toxicity based on sequence and structural parameters while recommending alternative amino acids to minimize toxicity. Analysis using this web-based tool for designed peptides indicated that peptides created for S and RdRp target proteins were non-toxic, supporting therapeutic development. However, toxicity was observed for the N protein model, which is understandable given that their design was based on optimization of a lead peptide (Plectasin) that itself exhibited toxicity. Importantly, for the selected analogue peptide A7, toxicity levels were lower compared to Plectasin. These findings underscore the importance of scaffold selection in peptide drug design and highlight opportunities for further optimization to minimize toxicity while preserving efficacy.

### 5.6. GRAVY Index and Its Interpretation

The Grand Average of Hydropathicity (GRAVY) index quantifies a peptide's hydrophobicity. A positive GRAVY value indicates a hydrophobic peptide, typically buried in protein cores or interacting with membranes. Conversely, a negative value signifies a hydrophilic peptide that is more soluble in aqueous environments (19). In this study, the designed S and RdRp inhibitors all exhibit negative GRAVY values, which is consistent with their required function as soluble, active inhibitors in a biological context.

### 5.7. Computational Limitations and Future Perspectives

Molecular docking was employed to identify the most effective peptides for inhibiting COVID-19 virulence proteins. While critical in drug discovery, molecular docking possesses significant limitations, as it does not consistently predict correct ligand binding modes due to algorithmic approximations of real-world interactions. Consequently, each docking run generates multiple docked poses ranked by docking scores. Factors that can accurately predict binding modes include small binding site dimensions, compact ligands, comprehensive receptor understanding, broader binding site constraints, and experienced practitioners. Challenges to accurate pose prediction include false binding sites due to receptor flexibility resulting in altered conformations, numerous tight-fitting deep pockets, and polar interaction directionality. The drug discovery process demands that molecules reach the market quickly to meet healthcare needs; therefore, molecular docking combined with MD can significantly facilitate this process, making it both cost-efficient and time-effective (39, 40).

The computational drug design pipeline — encompassing target identification, molecular docking, dynamics simulation, and safety assessment — offers a systematic approach for therapeutic development. This methodology enables:

Rapid candidate screening: Evaluation of thousands of potential inhibitors computationally

Structure-activity relationship elucidation: Understanding of molecular determinants of activity

Lead optimization: Rational design of improved analogues based on mechanistic insights

Risk assessment: Early identification of potential safety concerns (41, 42).

### 5.8. Molecular Dynamics Simulation Analysis

Ligand conformational stability is crucial for inhibitory action on target proteins, and MD simulation evaluation provides comprehensive examination of protein-ligand complex conformational landscapes under physiologically relevant conditions (41, 43). Molecular dynamics simulations in this study elucidated structural stability and binding dynamics of peptide inhibitors targeting SARS-CoV-2 proteins, with results interpreted in light of prior computational viral inhibition studies.

The W strand and EK1-S2 peptides bound to the spike (S) protein exhibited moderate flexibility (RMSD 0.5 - 0.8 nm) while maintaining receptor stability (RMSD < 0.3 nm), indicating adaptive binding reminiscent of natural protein-protein interactions. This behavior parallels observations with plant-derived RdRp inhibitors like SaikosaponinB2 (ΔGbinding = -42.43 kcal/mol) reported by Saha et al., where balanced rigidity-flexibility enhances inhibitory potential (35). In contrast, N-A7 and N-Plectasin complexes showed higher peptide mobility (RMSD 1.2-1.6 nm), analogous to suboptimal binding of Hesperidin (ΔGbinding = -22.72 kcal/mol) in the same study, suggesting that excessive conformational flexibility may compromise inhibitory efficacy. This finding emphasizes the importance of balanced structural dynamics in peptide inhibitor design. The RdRp-A5 peptide demonstrated superior conformational stability (RMSD: 0.8 - 1.0 nm) compared to parent sequence (Peptide 5), aligning with correlations between low RMSD and enhanced binding affinity in RdRp-inhibitor complexes reported by Saha et al. (35). This finding aligns with Kabra & Singh, whose machine-learning-designed peptides against SARS-CoV-2 Mpro emphasized the importance of targeting conserved, rigid domains (e.g., catalytic palm region of RdRp) with dynamically adaptable ligands.

These comparative results align with computational studies emphasizing the need for peptide inhibitors to harmonize structural plasticity with stable target engagement, a principle consistently validated across viral protein inhibition studies (36).

### 5.9. Conclusions

Peptide-based inhibitors targeting S, RdRp, and N receptor proteins show promise for treating SARS-CoV-2. Key factors affecting the effectiveness of these inhibitors include peptide length, amino acid composition, the formation of hydrophobic-hydrophobic and electrostatic bonds with the receptor, and the propensity for alpha-helical structure. These factors can enhance the binding affinity of peptides to the discussed proteins. This research utilized bioinformatics algorithms and web tools to develop a new peptide derived from specific lead peptides of each receptor, aiming to enhance alpha helicity for the lead peptide of the S receptor and to increase hydrophobic-hydrophobic and electrostatic bonds for the lead peptide targeting the RdRp receptor. Our results indicate that peptides W, A5, and A7 are the best new analogues for inhibiting proteins S, RdRp, and N, respectively. Based on the characteristics of these analogues, including their receptor specificity and superior docking results compared to lead peptides, they are presented as potential drug candidates for developing safe and effective SARS-CoV-2 treatments. Furthermore, applying bioinformatics algorithms to investigate structural changes resulting from mutated amino acid substitutions and the potential impact on the secondary structure of the designed peptides yields valuable insights for the rational design of peptide-based SARS-CoV-2 treatments. Further, in vitro and animal studies are recommended to validate these drug candidates as effective therapeutics.

ijpr-25-1-160762-s001.pdf

## Data Availability

The dataset presented in the study is available on request from the corresponding author during submission or after publication.
