# Supplementary material for: Innovative Peptide Therapeutics for SARS-CoV-2: Design, Docking, and Functional Analysis
Source: Iran J Pharm Res. 2026 Feb 15;25(1):e160762. doi: 10.5812/ijpr-160762 (PMC12933649; doi:10.5812/ijpr-160762)
Supplement: ijpr-25-1-160762-s001.pdf [file ijpr-25-1-160762-s001.pdf]

Appendix 1. The interaction between protein S receptor amino acids and EK1 peptide according to Delta-ACC

Amino acid from EK1 peptide	Amino acid in S receptor
LEU1002	VAL 963,A,6
	SER 967,A,17
ILE1005	LEU 959,A,6
	ASN 960,A,2
	VAL 963,A,28
VAL1007	ALA 956,A,9
	LEU 959,A,14
	ASN 960,A,22
	VAL 963,A,2
PHE1009	ASN 953,A,17
	ALA 956,A,7
	GLN 957,A,16
	ASN 960,A,9
LEU1010	LEU 948,A,3
	GLN 949,A,7
	VAL 952,A,15
	ASN 953,A,7
LEU1012	LEU 945,A,22
	LEU 948,A,3
	GLN 949,A,5
MET1016	ALA 942,A,18
	LEU 945,A,9
	GLY 946,A,14
	GLN 949,A,7
LEU1019	LEU 938,A,25
	THR 941,A,10
	ALA 942,A,7
	LEU 945,A,9
ILE1023	GLN 935,A,25
	LEU 938,A,6
	SER 939,A,15
	ALA 942,A,2

LEU1026	ILE 931,A,20
	ILE 934,A,12
	GLN 935,A,6
	LEU 938,A,8
TYR1030	ASN 928,A,23
	ILE 931,A,9
	GLY 932,A,8
	GLN 935,A,10
ILE1031	ILE 923,A,1
	ALA 924,A,21
	PHE 927,A,17
	ASN 928,A,4
LEU1033	TYR 917,A,2
	LYS 921,A,14
	ALA 924,A,3
	ASN 925,A,29
LEU1036	TYR 917,A,32
	GLN 920,A,23
	LYS 921,A,8
	ALA 924,A,8

Appendix 2. Results of the study of physicochemical properties, number of basic, acidic and neutral amino acids and hydrophobic amino acids for Lead peptide EK1 and its derived analogs for protein S receptor inhibition

NO	Name	GRAVY	Mw average g/mol	Theoretical pI (PH)	Charge at PH7	Acidic%	Basic%	Neutral%	Hydrophobic %
1	EK1	-0.43	4331.9	4.14	-5	27.78	13.89	13.89	44.44
2	E	-0.21	4331.94	4.07	-6	30.56	13.89	8.33	47.22
3	F	-0.21	4317.92	4.03	-6	30.56	13.89	8.33	47.22
4	G	-0.27	4289.86	4.07	-6	30.56	13.89	8.33	47.22
5	H	-0.12	4231.83	4.14	-5	27.78	13.89	8.33	50
6	I	-0.18	4197.77	4.07	-6	30.56	13.89	8.33	47.22
7	L	-0.2	4281.88	4.01	-7	33.33	13.89	5.56	47.22
8	O	-0.22	4308.91	4.07	-6	30.56	13.89	8.33	47.22
9	P	-0.22	4294.89	4.03	-6	30.56	13.89	8.33	47.22
10	S	-0.14	4267.86	4.07	-6	30.56	13.89	8.33	47.22
11	T	-0.14	4253.83	4.03	-6	30.56	13.89	8.33	47.22
12	U	-0.13	4225.82	4.03	-6	30.56	13.89	8.33	47.22
13	V	-0.13	4225.82	4.03	-6	30.56	13.89	8.33	47.22
14	W	-0.21	4271.9	4.27	-4	25	13.89	11.11	50
15	Y	0.01	4271.94	4.18	-5	27.78	13.89	5.56	52.78
16	Z	-0.01	4299.95	4.18	-5	27.78	13.89	5.56	52.78
17	1	-0.01	4285.92	4.14	-5	27.78	13.89	5.56	52.78
18	2	-0.03	4285.92	4.14	-5	27.78	13.89	5.56	52.78
19	3	-0/29	4299.91	4.18	-5	27.78	13. 89	8.33	50
20	4	-0.07	4298.96	4.18	-5	27.78	13.89	5.56	52.78
21	5	0.08	4240.93	4.27	-4	25	13.89	5.56	55.56
22	A	-0.43	4332.89	4.07	-6	30.56	13.89	11.11	44.44
23	B	-0.43	4318.86	4.03	-6	30.56	13.89	11.11	44.44
24	C	-0.37	4366.95	4.14	-5	27.78	13.89	11.11	47.22
25	D	-0.21	4330.96	4.14	-5	27.78	13.89	11.11	47.22
26	J	-0.13	4239.85	4.07	-6	30.56	13.89	8.33	47.22
27	K	-0.13	4239.85	4.07	-6	30.56	13.89	8.33	47.22
28	M	-0.2	4280.9	4.07	-6	30.56	13.89	8.33	47.22
29	N	-0.2	4280.9	4.07	-6	30.56	13.89	8.33	47.22

30	Q	-0.2	4266.87	4.03	-6	30.56	13.89	8.33	47.22
31	R	-0.2	4266.87	4.03	-6	30.56	13.89	8.33	47.22
32	X	-0.21	4272.88	4.18	-5	27.78	13.89	8.33	50
33	6	-0.15	4366	4.14	-5	27.78	13.89	8.33	50

Appendix 3: Results of the study of physicochemical properties and the number of basic, acidic, and neutral amino acids and hydrophobic amino acids for lead peptides number 4 and 5 and their derived analogs for inhibiting the RdRp protein receptor

NO	Name	GRAVY	Mw average g/mol	Theoretical pI (PH)	Charge at PH7	Acidic%	Basic%	Neutral%	Hydrophobic%
1	peptide 5	-2.53	488.54	10.09	1	25	50	0	25
2	A1	-2.53	502.56	10.09	1	25	50	0	25
3	A2	-2.35	511.53	7.88	0.1	25	50	0	25
4	A3	-3.78	568.62	10.09	1.1	25	75	0	0
5	A4	-3.85	546.57	7	0	50	50	0	0
6	A5	-4	574.58	7	0	50	50	0	0
7	A6	-2.68	530.58	10.88	1	25	50	0	25
8	A7	-3.68	555.54	5.22	`-0.9	50	50	0	0

Appendix 4: Results of the study of physicochemical properties, number of basic, acidic and neutral amino acids and hydrophobic amino acids for the lead peptide Plectasin and its derived analogs for the inhibition of the N-protein receptor

No	Name	GRAVY	Mw average g/mol	Theoretical pI (PH)	Charge at PH7	Acidic%	Basic%	Neutral%	Hydrophobic %
1	Plectasin	-0.7	4407.94	7.64	0.9	10	17.5	32.5	40
2	A1	-0.7	4421.97	7.64	0.9	10	17.5	32.5	40
3	A2	-0.71	4398.93	7.64	0.9	10	17.5	32.5	40
4	A3	-0.76	4379.89	7.64	0.9	10	17.5	32.5	40
5	A4	-0.71	4384.91	7.64	0.9	10	17.5	32.5	40
6	A5	-0.77	4370.88	7.64	0.9	10	17.5	32.5	40
7	A6	-0.86	4409.92	7.64	0.9	10	17.5	32.5	40
8	A7	-0.78	4398.89	7.64	0.9	10	17.5	32.5	40
9	A8	-0.73	4322.84	7.64	0.9	10	17.5	32.5	40
10	A9	-0.75	4350.85	7.64	0.9	10	17.5	32.5	40
11	A10	-0.87	4437.93	7.64	0.9	10	17.5	32.5	40
12	A11	-0.81	4440.98	8.01	1.9	10	20	30	40
13	A12	-0.78	4399.88	6.92	-0.1	12.5	17.5	30	40
14	A13	-0.78	4384.87	7.64	0.9	10	17.5	32.5	40
15	A14	-0.77	4356.85	7.64	0.9	10	17.5	32.5	40
16	A15	-0.72	4412.92	7.64	0.9	10	17.5	32.5	40
17	A16	-0.79	4377.92	8.01	1.9	10	20	32.5	37.5
18	A17	-0.76	4350.85	6.92	-0.1	12.5	17.5	32.5	37.5
19	A18	-0.6	4368.91	7.64	0.9	10	17.5	32.5	40
20	A19	-0.71	4318.85	7.64	0.9	10	17.5	35	37.5
21	A20	-0.58	4334.89	7.64	0.9	10	17.5	32.5	40
